# Detection of Pin Failure in Carbon Fiber Composites Using the Electro-Mechanical Impedance Method

**DOI:** 10.3390/s20133732

**Published:** 2020-07-03

**Authors:** Jochen Moll, Matthias Schmidt, Johannes Käsgen, Jörg Mehldau, Marcel Bücker, Felix Haupt

**Affiliations:** 1 Department of Physics, Goethe University Frankfurt, D-60438 Frankfurt am Main, Germany; 2 Fraunhofer Institut LBF, 64289 Darmstadt, Germany; matthias.schmidt@lbf.fraunhofer.de (M.S.); johannes.kaesgen@lbf.fraunhofer.de (J.K.); 3 Becker Marine Systems GmbH, 21079 Hamburg, Germany; jme@becker-marine-systems.com; 4 Institut für Verbundwerkstoffe GmbH, 67663 Kaiserslautern, Germany; Marcel.Buecker@ivw.uni-kl.de; 5 CCOR—Schäfer MWN GmbH, 71272 Renningen, Germany; F.Haupt@ccor.com

**Keywords:** structural health monitoring, damage detection, composites, electro-mechanical impedance, smart sensor systems

## Abstract

This paper presents a proof of concept for simultaneous load and structural health monitoring of a hybrid carbon fiber rudder stock sample consisting of carbon fiber composite and metallic parts in order to demonstrate smart sensors in the context of maritime systems. Therefore, a strain gauge is used to assess bending loads during quasi-static laboratory testing. In addition, six piezoelectric transducers are placed around the circumference of the tubular structure for damage detection based on the electro-mechanical impedance (EMI) method. A damage indicator has been defined that exploits the real and imaginary parts of the admittance for the detection of pin failure in the rudder stock. In particular, higher frequencies in the EMI spectrum contain valuable information about damage. Finally, the information about damage and load are merged in a cluster analysis enabling damage detection under load.

## 1. Introduction

The electro-mechanical impedance (EMI) method is widely used in structural health monitoring (SHM) applications to detect damage in isotropic and composite materials [1,2]. The underlying idea proposed by Liang et al. [3] is based on the fact that the electrical impedance of a piezoelectric patch is linked to the mechanical impedance of the structure. A structural defect, e.g., a delamination or crack, can be detected by a change in the impedance characteristics (or in the admittance as its inverse).

In recent years, the EMI method has been applied successfully on composite materials. An example is the work presented by Wandowski et al. [4] who used the EMI method to detect delamination in carbon fiber reinforced materials. Effects related to temperature were compensated by a correlation-based method. Another successful application of the EMI technique in composite materials was reported in [5], in which resonance frequencies of piezoelectric transducers were used to improve damage detection performance. Impact damage detection in composite structures have been presented by Sharif-Khodaei et al. [6] using numerical and experimental investigations. The authors also considered several parameters of the sensing technique, including environmental effects, frequency sweep, severity of damage and the location of damage. More recently, the EMI-method was applied for damage detection in coupons constructed as fiber reinforced materials [7]. In that work, the damage detection performance was studied for different attachment methods of piezoelectric transducers. A portable system for impedance-based testing of composite structures is presented in [8].

This work aims at smart sensor systems for damage detection in maritime structures that require an extremely high reliability so that methods are crucially needed to continuously monitor structural conditions. In the literature, several SHM systems are presented for damage detection in ship structures: a first example is the work proposed by Osaka et al. [9], who integrated an SHM methodology in life-cycle performance assessment. Moreover, a model-based approach for SHM of ship hulls is demonstrated in [10]. Zhirnov et al. [11] followed a different approach and combined global and local SHM techniques for structural assessment of icebreakers. A wireless SHM system for submarine structures is presented in [12]. More recently, Kefal [13] investigated a structural monitoring approach of cylindrical marine structures using dense, sparse and very sparse sensor deployments.

Related works also come from the field of load monitoring systems of maritime components. In most cases, resistive or optical (fiber Bragg grating) strain gauges are used for this task [14]. In addition, accelerometers have been used in [15] to detect global resonant behavior (whipping). Such load monitoring approaches enable the estimation of the structure’s remaining life time and provide immediate feedback to the operator to allow real-time changes in maneuvering to minimize overstressing [15]. Load monitoring is particularly important for ships moving in polar regions. The estimation of ice loads acting on the ship’s hull was investigated by Leira et al. [16] by means of strain measurements. In addition, Orihara et al. [17] have studied loading effects in large merchant ships and compared the results with theoretical predictions.

**Figure 1:**
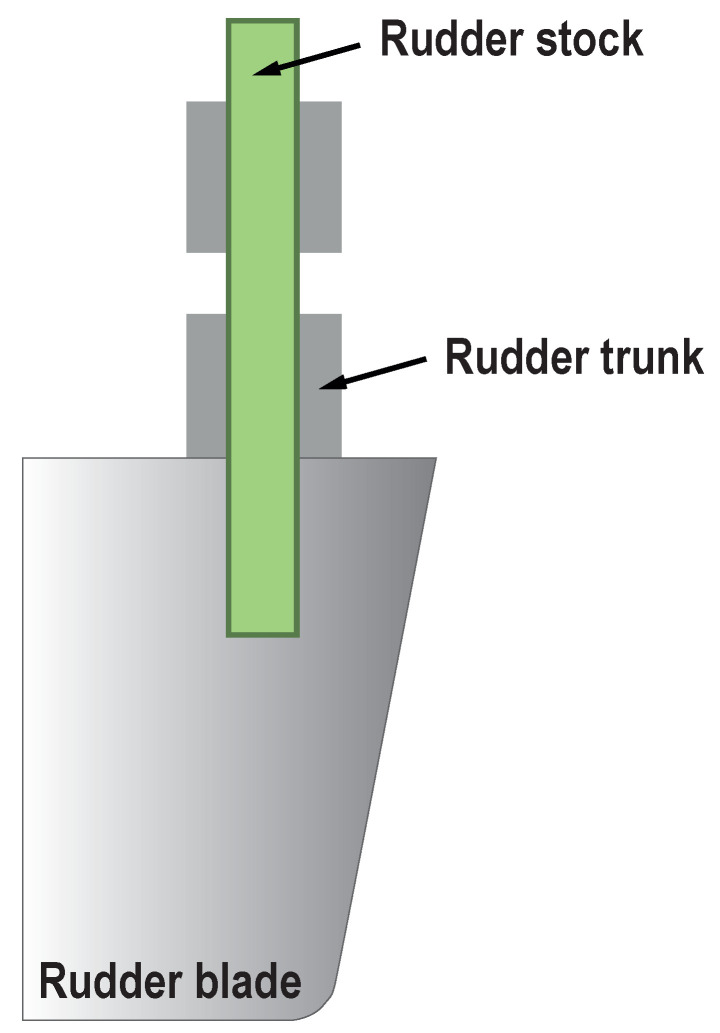
Illustration of the rudder stock as part of the rudder system.

Here, we focus on a rudder stock as part of a rudder system, as depicted in Figure 1. Similarly to recent developments in the aviation industry, more fiber reinforced materials are used nowadays. This leads to weight savings in the order of 30–50% while keeping structural stiffness. As a result, fuel consumption during ship operation can be optimized. The rudder stock in this work has a hybrid mechanical design including carbon fiber reinforced plastic (CFRP) and metallic parts. In previous works, we have successfully applied the EMI method to thick CFRP components during tensile tests [18]. Instead of coupon testing, we present here experimental results of a rudder stock demonstrator in a dedicated test stand. The goal is to show the damage detection capability under load combined with structural monitoring based on the EMI method. A particular focus is on the failure detection of metallic pins connecting the steel and CFRP parts.

The remainder of the paper is organized in the following way: Section 2 describes the hybrid rudder stock design and the experimental setup. After that, Section 3 shows the damage detection results using a damage indicator followed by a cluster analysis. Finally, conclusions are drawn in Section 4.

## 2. Description of the Experimental Setup

**Figure 2:**
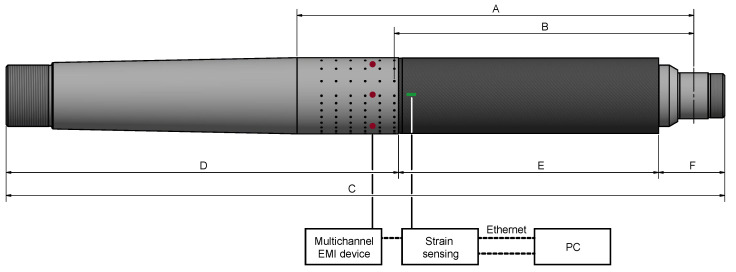
Geometry of the rudder stock, the sensor locations and an illustration of the data acquisition procedure.

**Figure 3:**
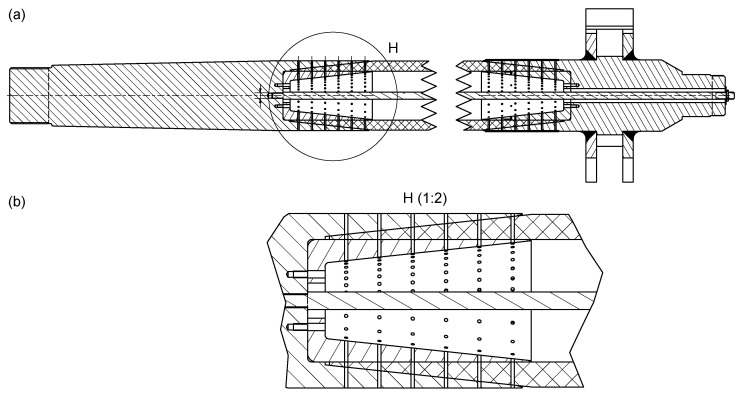
(**a**) Technical drawing of the rudder stock; (**b**) close-up of the interface between steel and CFRP, including pin locations arranged in six pin rows.

The rudder stock is made of a material combination of CFRP and steel. The main reason for this design is that the new rudder stock should be exchangeable with the state of the art full steel rudder stock. In these rudder stocks, the rudder blade and the steering gear, on the other hand, are mounted to the rudder stock with conical press fits. So couplings of this kind should not be touched, and thereby the other parts do not need any changes in construction. But a conical press fit due to the locally applied high surface pressures cannot be realized in CFRP. Due to the relatively low stiffness and strength in radial direction and creeping effects of the polymer resin, a CFRP cone could not withstand the surface loads permanently. As a solution, the new rudder stock is made of three components: keep steel as the material for both ends of the rudder stock and couple these two steel parts with a middle section made of CFRP (see Figure 2 and Figure 3). Due to the very high torsional and bending loads in the application case, the bonding between the two steel components and the CFRP part is critical. A scaled and adapted prototype of the rudder stock with one of the two bonding regions is shown in Figure 2 with major dimensions listed in Table 1. A double-lapped shear connection with several hardened steel pins between the steel end and the CFRP-section was chosen to achieve a fail-safe connection. This kind of connection is able to transfer all loads within a very short coupling length, so that the heavy and relatively expensive steel parts can be kept very short. Overall, six pin rows in circumferential direction are needed wherein the pins are pointing in the radial direction, as shown in Figure 3. In the testing prototype, these circumferential rings of pins are divided into four equal sections: two opposite ones with full pin density; the other two with half the pin density to aim at a different load case by turning the rudder stock in the rig by 90 degrees. To identify possible load and damage sources, a failure mode and effect analysis (FMEA) was made as basis for the monitoring methodology. The load cases derived for the laboratory investigation of the rudder stock are the result of the FMEA, FE simulation and data records from past ship movements with occurring rudder forces. With the rudder stock scaled to 1:10, the load cases range from nominal bending load of 282 kN to break load of 1128 kN.

**Figure 4:**
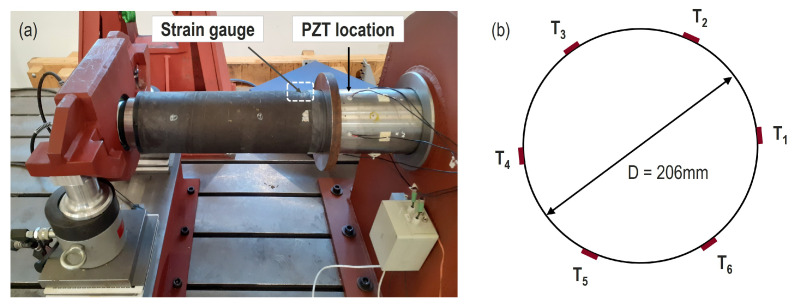
(**a**) Photo of the experimental setup showing the rudder stock in the test stand. (**b**) Positions of the piezoelectric transducers around the circumference.

**Figure 5:**
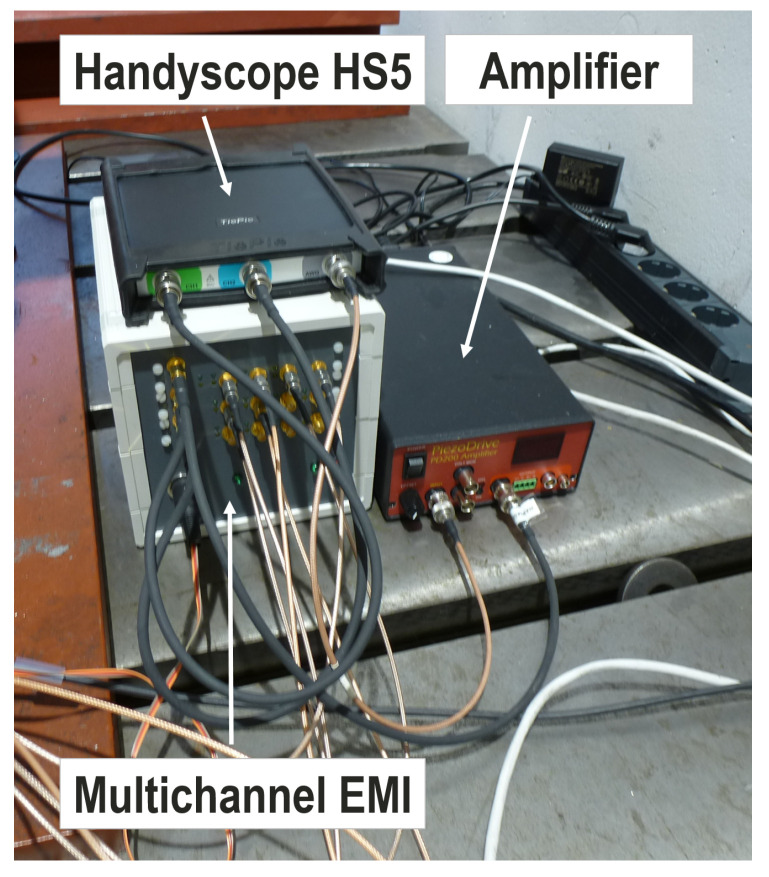
Photo of the electro-mechanical impedance (EMI) subsystem, including the multichannel EMI device, a Handscope HS5 for signal generation and A/D conversion and an ultrasound amplifier.

Figure 4 shows the test stand and the rudder stock sample for the laboratory investigation at a constant temperature of $21\;{}^{{}^{\circ}}C$. The hydraulic cylinders can provide forces up to 1900 kN to create bending load. The data acquisition and monitoring are based on embedded PCs (host and target) utilizing an EtherCAT sensor network with strain gauges (HBM 1-LI66-10/350), thermocouples and piezo patches (PIC 255).The circular piezoelectric transducers have a diameter of 10 mm and thickness of 0.25 mm. The frequency characteristics of those transducers have been investigated, for example, in [19]. The transducers have been placed over the circumference of the rudder stock with an angular spacing of about $60^{{}^{\circ}}$ (see right part of Figure 4). The attachment was realized by HBM X60 glue. The EMI subsystem is shown in Figure 5, consisting of the multichannel EMI unit, a Handyscope HS5 (Tiepie Engineering) and an ultrasound amplifier PD200 (PiezoDrive Ltd.). A detailed description of the multichannel device can be found in [18], enabling high frequencies (up to 1 MHz) and high currents (up to 2 A) so that highly attenuated materials such as the proposed CFRP rudder stock can be inspected. In this work, the frequency range of interest was 25 kHz to 500 kHz.

## 3. Results

**Figure 6:**
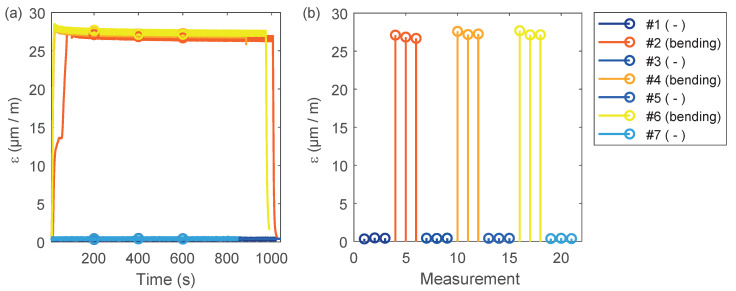
(**a**) Static strain that is proportional to the current bending loads as a function of time. (**b**) Modified representation of (a) showing the alternating nature of the applied loading. Three EMI measurements were successively recorded during one loading cycle.

This work uses static alternating bending loads applied to the rudder stock to model reference conditions in the laboratory as realistically as possible. The varying operational conditions can be managed in practical SHM systems by means of a classification technique using multiple reference conditions that are learned during a training phase [20]. The idea of reference conditions is motivated by the fact that the current measurement can be compared with a baseline condition in a meaningful way. Relative changes in the signals can be attributed to structural damage. A plot of the alternating load is shown in Figure 6b. Please note the color coding of the curves will also be used later. Here, blue indicates the unloaded structure and warm colors represent loaded structural conditions.

Similarly to related works, compare, for example [4,8,21,22], we consider a damage indicator (DI) to evaluate structural conditions. The DIs should have small values for the intact structure and increased values when damage occurs. Here, we consider the root-mean-square deviation (RMSD) to compare the admittance measurement $Y_{i}$ to the baseline state $Y_{b}$. We consider the real and imaginary parts of the admittance for damage assessment:

(1) $$DI^{real}\left[Y_{i},Y_{b}\right]=\sqrt{\frac{1}{n}\sum_{j=1}^{n}{\left[ℜ\left(Y_{i}\left(f_{j}\right)\right)-ℜ\left(Y_{b}\left(f_{j}\right)\right)\right]}^{2}}$$

(2) $$DI^{imag}\left[Y_{i},Y_{b}\right]=\sqrt{\frac{1}{n}\sum_{j=1}^{n}{\left[ℑ\left(Y_{i}\left(f_{j}\right)\right)-ℜ\left(Y_{b}\left(f_{j}\right)\right)\right]}^{2}}.$$

In these equations, *f* denotes frequency and *n* the number of measurement points.

**Figure 7:**
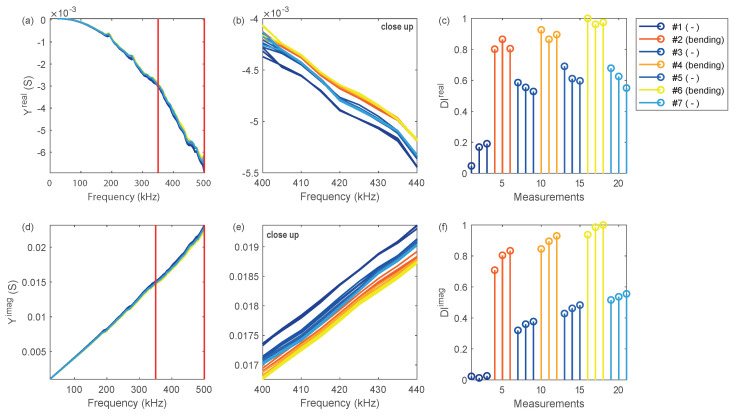
EMI analysis for transducer $T_{3}$: (**a**) real part of the admittance; (**b**) real part of the admittance (close up); (**c**) $DI^{real}$ for different loading conditions; (**d**) imaginary part of the admittance; (**e**) imaginary part of the admittance (close up); (**f**) $DI^{imag}$ for different loading conditions. All DIs were calculated for the frequency band of 350–500 kHz showing a slightly improved contrast between intact and damaged structure compared to the full frequency spectrum.

Figure 7a,b shows the development of the real part of the admittance as a function of frequency for transducer $T_{3}$. A clear offset between the curves can be seen comparing the unloaded and loaded structure. The DIs for these measurements are shown in Figure 7c. We find that the DI of the undamaged structure (measurements 1 to 3) is relatively small and increases significantly through the application of the bending load (measurements 4 to 6). Next, the structure is unloaded again, and the three subsequent EMI measurements show a similar value, but at an increased level compared with the baseline measurements. This means that damage in form of a pin failure already occurred after the first loading. The periodic pattern in the DI curve can be explained by the alternating change of load and unload.

Similarly, the imaginary part of the DI shows an offset between the unloaded and the loaded rudder stock. The corresponding DI curve confirms the previous findings in the sense that the DI remains at an increased level after the first loading cycle. In addition, we see that $DI^{imag}$ further increases after each loading cycle, showing independent diagnostic information. This observation will be exploited subsequently in a cluster analysis.

**Figure 8:**
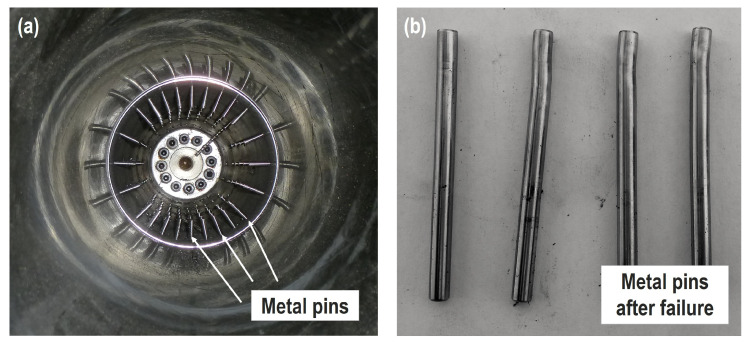
(**a**) View in the rudder stock showing the distribution of metal pins (higher pin density in vertical direction); (**b**) deformed metal pins after failure.

After finalizing the measurements, the rudder stock was carefully disassembled to evaluate the condition of the metal pins. Figure 8a shows a photo of the inner part of the rudder stock with the distribution of the metal pins. It was found that many metal pins were deformed around the circumference, as shown in Figure 8b, due to the application of the bending load. This confirms that the proposed EMI technique is able to detect this type of damage.

**Figure 9:**
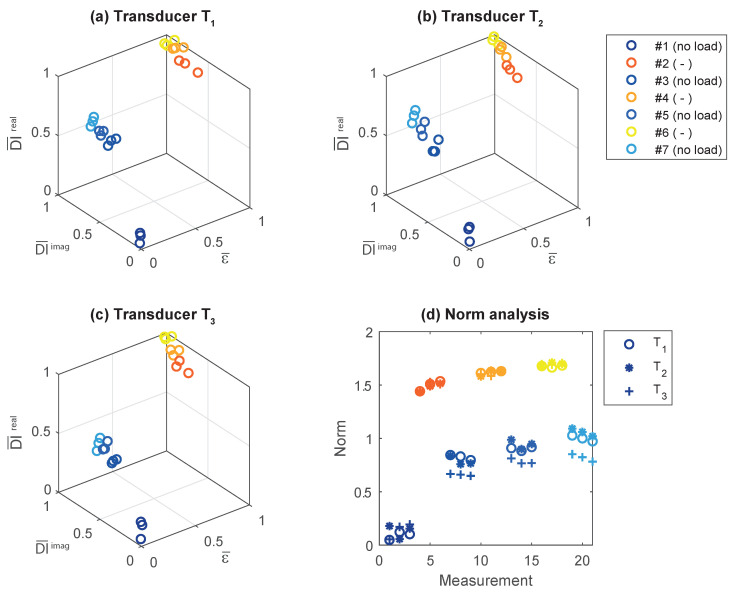
(**a**–**c**) Cluster analysis for transducers $T_{1}$–$T_{3}$ where all quantities have been normalized to the interval [0, 1]. (**d**) Norm relative to the coordinate origin (0,0,0). All piezoelectric transducers contain similar information about damage. This is plausible, because pin failures occurred around the circumference of the specimen. The proposed cluster analysis enables a clear distinction between the different loading and damage conditions.

In a next step, we performed a cluster analysis exemplarily for the transducers $T_{1}$ to $T_{3}$ taking into account the bending load in terms of the normalized strain and the real and imaginary parts of the DI in normalized form (see Figure 9). It is interesting to see that clusters emerge where three subsequent measurements belong to the same cluster. A discrimination between the different structural conditions can be found, taking into account the strain information. Finally, the Euclidean norm with respect to the coordinate origin (0,0,0) is computed. The resulting curve for the norm is depicted in Figure 7d, showing that all piezoelectric transducers carry similar diagnostic information about the pin failures. This is plausible, because pin failures occurred around the circumference of the specimen.

## 4. Conclusions

This work successfully demonstrated the application of the electromechanical impedance (EMI) method for damage detection in a steel-composite rudder stock sample. The proof of concept used six piezoelectric transducers aligned on the circumference of the rudder stock for EMI-based testing. In addition, strain gauges were used to assess static bending loads. The EMI method in the frequency range up to 500 kHz enabled the detection of pin failure as a highly loaded part of the rudder stock design. In a next step, the promising results obtained in this study need to be further investigated with a higher number of rudder stock samples and dynamic loading.

## Figures and Tables

**Table 1:** Detailed description of the dimensions shown in Figure 2.

Code	Description	Value
A	attack point actuator to mounting point	1120 mm
B	attack point actuator to first row of pins	900 mm
C	total length	2000 mm
D	length of steel mounting	1025 mm
E	length of CFRP tube	800 mm
F	outside length of actuator mounting	173 mm

## Abbreviations

The following abbreviations are used in this manuscript:

EMI	electro-mechanical impedance
SHM	structural health monitoring
